# 1,3,3-Trimethyl-1,2,3,4-tetra­hydro­pyrido[1,2-*a*]benzimidazol-1-ol

**DOI:** 10.1107/S1600536810024487

**Published:** 2010-06-26

**Authors:** Sayed Hasan Mehdi, Rokiah Hashim, Raza Murad Ghalib, Chin Sing Yeap, Hoong-Kun Fun

**Affiliations:** a School of Industrial Technology, Universiti Sains Malaysia, 11800 USM, Penang, Malaysia; b X-ray Crystallography Unit, School of Physics, Universiti Sains Malaysia, 11800 USM, Penang, Malaysia

## Abstract

In the title compound, C_14_H_18_N_2_O, the benzimidazole grouping is close to planar, with a maximum deviation of 0.042 Å; the six-membered non-aromatic ring adopts an envelope conformation. In the crystal structure, mol­ecules are linked into infinite sheets lying parallel to the *bc* plane by O—H⋯N and C—H⋯O hydrogen bonds.

## Related literature

For applications of benzimidazole derivatives, see: Horton *et al.* (2003 ▶); Insuasty *et al.* (2008*a* ▶,*b* ▶). For the preparation of the title compound, see: Grech *et al.* (1994 ▶). For ring conformations, see Cremer & Pople (1975 ▶). For the stability of the temperature controller used for the data collection, see: Cosier & Glazer (1986 ▶).
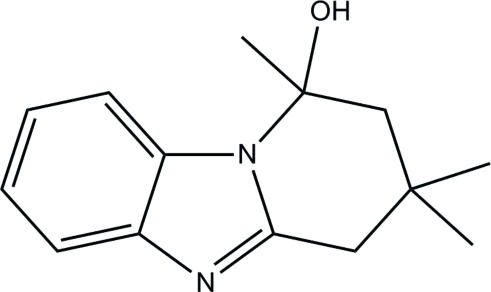


## Experimental

### 

#### Crystal data

C_14_H_18_N_2_O
*M* *_r_* = 230.30
Monoclinic, 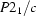

*a* = 9.615 (5) Å
*b* = 8.194 (4) Å
*c* = 15.965 (8) Å
β = 99.601 (12)°
*V* = 1240.2 (11) Å^3^
*Z* = 4
Mo *K*α radiation
μ = 0.08 mm^−1^
*T* = 100 K
0.38 × 0.12 × 0.07 mm

#### Data collection

Bruker APEXII DUO CCD diffractometer
Absorption correction: multi-scan (*SADABS*; Bruker, 2009 ▶) *T*_min_ = 0.971, *T*_max_ = 0.995
13460 measured reflections
3597 independent reflections
2612 reflections with *I* > 2σ(*I*)
*R*_int_ = 0.052

#### Refinement

*R*[*F* ^2^ > 2σ(*F* ^2^)] = 0.046
*wR*(*F* ^2^) = 0.117
*S* = 1.02
3597 reflections
226 parameters
All H-atom parameters refined
Δρ_max_ = 0.33 e Å^−3^
Δρ_min_ = −0.25 e Å^−3^

### 

Data collection: *APEX2* (Bruker, 2009 ▶); cell refinement: *SAINT* (Bruker, 2009 ▶); data reduction: *SAINT*; program(s) used to solve structure: *SHELXTL* (Sheldrick, 2008 ▶); program(s) used to refine structure: *SHELXTL*; molecular graphics: *SHELXTL*; software used to prepare material for publication: *SHELXTL* and *PLATON* (Spek, 2009 ▶).

## Supplementary Material

Crystal structure: contains datablocks global, I. DOI: 10.1107/S1600536810024487/hb5508sup1.cif

Structure factors: contains datablocks I. DOI: 10.1107/S1600536810024487/hb5508Isup2.hkl

Additional supplementary materials:  crystallographic information; 3D view; checkCIF report

## Figures and Tables

**Table 1:** Hydrogen-bond geometry (Å, °)

*D*—H⋯*A*	*D*—H	H⋯*A*	*D*⋯*A*	*D*—H⋯*A*
O1—H1*O*1⋯N1^i^	0.97 (2)	1.84 (2)	2.803 (2)	174 (2)
C5—H5*A*⋯O1^ii^	0.962 (15)	2.499 (15)	3.216 (2)	131.3 (11)

Symmetry codes: (i) 
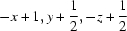
; (ii) 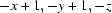.

**Fig. 1.**
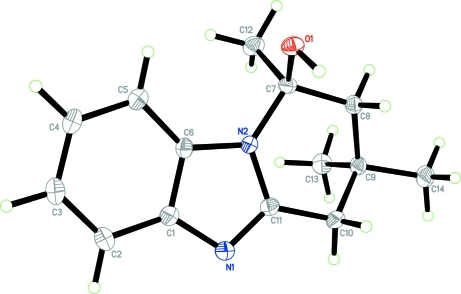
The molecular structure of (I) with 50% probability ellipsoids for non-H atoms.

**Fig. 2.**
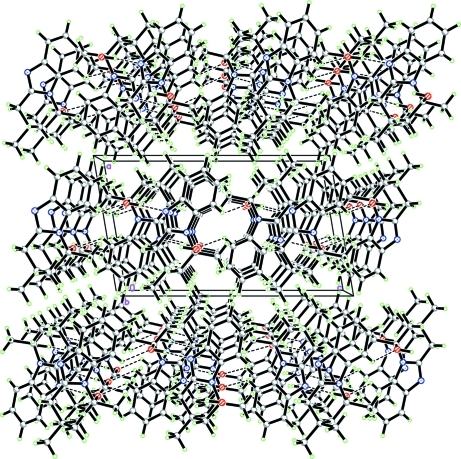
The crystal packing of (I), viewed down the b axis, showing the molecules linked into sheets lying parallel to bc. Intermolecular hydrogen bonds are shown as dashed lines.

## supplementary crystallographic information

### Comment

Benzimidazole derivatives are an important class of bioactive molecules and are well known due to their wide range of pharmacological activities as an anti-ulcers, anti-hypertensive, anti-viral, anti-fungal, anti-cancer, and anti-histaminic (Horton *et al.*, 2003; Insuasty *et al.*, 2008*a*, *b*) agents. Here we report the synthesis and the crystal structure of title compound.

In the title compound (Fig. 1), the benzimidazole group is essentially coplanar (N1/C1–C6/N2/C11) with the maximum deviation of 0.042 Å at atom C6. The N2/C7–C11 ring adopts an envelope conformation with Q=0.4933 (14) Å, θ=52.81 (15)° and φ=182.3 (2)° (Cremer & Pople, 1975).

In the crystal structure, the molecules are linked into infinite two-dimensional planes parallel to *bc* plane by the intermolecular O1—H1O1···N1 and C5—H5A···O1 hydrogen bonds (Fig. 2, Table 1).

### Experimental

A mixture of *o*-phenylenediamine (0.108 g m) and dimedone (0.140 g m) in molar ratio 1:1 was refluxed in a mixture of acetic acid-ethanol (1:1 *v*/*v*) for 3 h (Grech *et al.*, 1994). The reaction mixture was dried on rotavapor at low pressure and further fractionated successively with diethyl ether, chloroform and ethanol. The ethanol fraction was dried on rotavapor and the dry mass so obtained was crystallized in methanol:chloroform (1:1) mixture to give yellow needles of (I) (55%, m.p. 451 K).

### Refinement

All hydrogen atoms were located from the difference Fourier map and was refined freely.

### Crystal data

**Table:**

C_14_H_18_N_2_O	*F*(000) = 496
*M**_r_* = 230.30	*D*_x_ = 1.233 Mg m^−^^3^
Monoclinic, *P*2_1_/*c*	Mo *K*α radiation, λ = 0.71073 Å
Hall symbol: -P 2ybc	Cell parameters from 2162 reflections
*a* = 9.615 (5) Å	θ = 3.1–29.6°
*b* = 8.194 (4) Å	µ = 0.08 mm^−^^1^
*c* = 15.965 (8) Å	*T* = 100 K
β = 99.601 (12)°	Needle, yellow
*V* = 1240.2 (11) Å^3^	0.38 × 0.12 × 0.07 mm
*Z* = 4	

### Data collection

**Table:**

Bruker APEXII DUO CCD diffractometer	3597 independent reflections
Radiation source: fine-focus sealed tube	2612 reflections with *I* > 2σ(*I*)
graphite	*R*_int_ = 0.052
φ and ω scans	θ_max_ = 30.0°, θ_min_ = 2.6°
Absorption correction: multi-scan (*SADABS*; Bruker, 2009)	*h* = −13→13
*T*_min_ = 0.971, *T*_max_ = 0.995	*k* = −11→10
13460 measured reflections	*l* = −22→18

### Refinement

**Table:**

Refinement on *F*^2^	Primary atom site location: structure-invariant direct methods
Least-squares matrix: full	Secondary atom site location: difference Fourier map
*R*[*F*^2^ > 2σ(*F*^2^)] = 0.046	Hydrogen site location: inferred from neighbouring sites
*wR*(*F*^2^) = 0.117	All H-atom parameters refined
*S* = 1.02	*w* = 1/[σ^2^(*F*_o_^2^) + (0.0528*P*)^2^ + 0.2357*P*] where *P* = (*F*_o_^2^ + 2*F*_c_^2^)/3
3597 reflections	(Δ/σ)_max_ < 0.001
226 parameters	Δρ_max_ = 0.33 e Å^−^^3^
0 restraints	Δρ_min_ = −0.25 e Å^−^^3^

### Special details

**Table:**

Experimental. The crystal was placed in the cold stream of an Oxford Cryosystems Cobra open-flow nitrogen cryostat (Cosier & Glazer, 1986) operating at 100.0 (1) K.
Geometry. All e.s.d.'s (except the e.s.d. in the dihedral angle between two l.s. planes) are estimated using the full covariance matrix. The cell e.s.d.'s are taken into account individually in the estimation of e.s.d.'s in distances, angles and torsion angles; correlations between e.s.d.'s in cell parameters are only used when they are defined by crystal symmetry. An approximate (isotropic) treatment of cell e.s.d.'s is used for estimating e.s.d.'s involving l.s. planes.
Refinement. Refinement of *F*^2^ against ALL reflections. The weighted *R*-factor *wR* and goodness of fit *S* are based on *F*^2^, conventional *R*-factors *R* are based on *F*, with *F* set to zero for negative *F*^2^. The threshold expression of *F*^2^ > σ(*F*^2^) is used only for calculating *R*-factors(gt) *etc*. and is not relevant to the choice of reflections for refinement. *R*-factors based on *F*^2^ are statistically about twice as large as those based on *F*, and *R*- factors based on ALL data will be even larger.

### Fractional atomic coordinates and isotropic or equivalent isotropic displacement parameters (Å^2^)

**Table:**

	*x*	*y*	*z*	*U*_iso_*/*U*_eq_	
O1	0.65445 (9)	0.44319 (11)	0.12156 (6)	0.0178 (2)	
N1	0.40305 (10)	0.05504 (14)	0.22183 (7)	0.0176 (2)	
N2	0.54560 (10)	0.19700 (13)	0.15038 (6)	0.0148 (2)	
C1	0.32424 (12)	0.11377 (16)	0.14618 (8)	0.0160 (3)	
C2	0.17999 (13)	0.09810 (17)	0.11474 (9)	0.0199 (3)	
C3	0.12707 (13)	0.17745 (18)	0.03945 (9)	0.0215 (3)	
C4	0.21420 (13)	0.27314 (18)	−0.00320 (8)	0.0199 (3)	
C5	0.35783 (13)	0.28830 (17)	0.02639 (8)	0.0179 (3)	
C6	0.41136 (12)	0.20479 (15)	0.10110 (8)	0.0152 (2)	
C7	0.67647 (12)	0.27365 (16)	0.13019 (8)	0.0153 (3)	
C8	0.79944 (12)	0.23815 (16)	0.20337 (8)	0.0164 (3)	
C9	0.79390 (12)	0.07980 (16)	0.25468 (8)	0.0165 (3)	
C10	0.65512 (13)	0.08393 (18)	0.29055 (8)	0.0186 (3)	
C11	0.53240 (12)	0.10891 (16)	0.22157 (8)	0.0157 (3)	
C12	0.70543 (14)	0.21133 (19)	0.04460 (9)	0.0208 (3)	
C13	0.80018 (14)	−0.07437 (18)	0.20160 (9)	0.0218 (3)	
C14	0.91813 (13)	0.08019 (19)	0.32872 (9)	0.0224 (3)	
H1A	0.0269 (15)	0.1672 (18)	0.0155 (9)	0.020 (4)*	
H2A	0.1203 (16)	0.033 (2)	0.1467 (10)	0.026 (4)*	
H4A	0.1727 (15)	0.3345 (18)	−0.0553 (9)	0.016 (4)*	
H5A	0.4140 (15)	0.3569 (19)	−0.0037 (9)	0.017 (4)*	
H8A	0.8923 (16)	0.241 (2)	0.1794 (10)	0.025 (4)*	
H8B	0.8028 (15)	0.334 (2)	0.2438 (10)	0.025 (4)*	
H10A	0.6384 (15)	−0.0185 (19)	0.3209 (9)	0.018 (4)*	
H10B	0.6583 (16)	0.177 (2)	0.3319 (10)	0.024 (4)*	
H12A	0.7260 (17)	0.095 (2)	0.0457 (11)	0.030 (4)*	
H12B	0.7853 (17)	0.279 (2)	0.0300 (11)	0.032 (4)*	
H12C	0.6228 (18)	0.233 (2)	−0.0008 (11)	0.034 (5)*	
H13A	0.7168 (16)	−0.0878 (19)	0.1571 (10)	0.024 (4)*	
H13B	0.8059 (15)	−0.171 (2)	0.2371 (10)	0.024 (4)*	
H13C	0.8856 (17)	−0.074 (2)	0.1725 (11)	0.032 (4)*	
H14A	0.9187 (16)	0.180 (2)	0.3648 (10)	0.028 (4)*	
H14B	0.9107 (17)	−0.017 (2)	0.3658 (11)	0.034 (5)*	
H14C	1.0108 (18)	0.082 (2)	0.3068 (11)	0.036 (5)*	
H1O1	0.640 (2)	0.487 (3)	0.1756 (13)	0.050 (6)*	

### Atomic displacement parameters (Å^2^)

**Table:**

	*U*^11^	*U*^22^	*U*^33^	*U*^12^	*U*^13^	*U*^23^
O1	0.0215 (4)	0.0149 (5)	0.0175 (5)	0.0005 (3)	0.0047 (3)	0.0024 (4)
N1	0.0167 (5)	0.0195 (6)	0.0166 (5)	−0.0010 (4)	0.0028 (4)	0.0018 (4)
N2	0.0138 (4)	0.0170 (5)	0.0130 (5)	−0.0010 (4)	0.0009 (4)	0.0014 (4)
C1	0.0169 (5)	0.0153 (6)	0.0158 (6)	0.0004 (4)	0.0024 (4)	−0.0007 (5)
C2	0.0155 (5)	0.0206 (7)	0.0232 (7)	−0.0010 (5)	0.0020 (5)	−0.0017 (6)
C3	0.0171 (6)	0.0229 (7)	0.0229 (7)	0.0009 (5)	−0.0014 (5)	−0.0041 (6)
C4	0.0208 (6)	0.0210 (7)	0.0164 (6)	0.0036 (5)	−0.0018 (5)	−0.0009 (5)
C5	0.0202 (6)	0.0173 (7)	0.0157 (6)	0.0013 (5)	0.0014 (5)	0.0001 (5)
C6	0.0148 (5)	0.0160 (6)	0.0144 (6)	0.0001 (4)	0.0010 (4)	−0.0019 (5)
C7	0.0154 (5)	0.0150 (6)	0.0157 (6)	−0.0013 (4)	0.0036 (4)	0.0021 (5)
C8	0.0157 (5)	0.0162 (6)	0.0167 (6)	−0.0007 (4)	0.0007 (4)	0.0004 (5)
C9	0.0145 (5)	0.0169 (6)	0.0170 (6)	0.0002 (4)	0.0000 (4)	0.0018 (5)
C10	0.0176 (5)	0.0229 (7)	0.0147 (6)	−0.0007 (5)	0.0012 (4)	0.0041 (6)
C11	0.0168 (5)	0.0153 (6)	0.0150 (6)	0.0006 (4)	0.0030 (4)	0.0017 (5)
C12	0.0223 (6)	0.0249 (8)	0.0164 (6)	0.0014 (5)	0.0067 (5)	−0.0008 (6)
C13	0.0230 (6)	0.0177 (7)	0.0241 (7)	0.0018 (5)	0.0017 (5)	0.0002 (6)
C14	0.0185 (6)	0.0244 (8)	0.0220 (7)	0.0007 (5)	−0.0030 (5)	0.0038 (6)

### Geometric parameters (Å, °)

**Table:**

O1—C7	1.4085 (17)	C8—C9	1.5400 (19)
O1—H1O1	0.96 (2)	C8—H8A	1.029 (15)
N1—C11	1.3203 (16)	C8—H8B	1.012 (16)
N1—C1	1.3997 (17)	C9—C13	1.528 (2)
N2—C11	1.3700 (17)	C9—C14	1.5340 (18)
N2—C6	1.3965 (16)	C9—C10	1.5378 (18)
N2—C7	1.4890 (16)	C10—C11	1.4878 (18)
C1—C2	1.4000 (18)	C10—H10A	0.995 (16)
C1—C6	1.4060 (18)	C10—H10B	1.004 (16)
C2—C3	1.387 (2)	C12—H12A	0.976 (18)
C2—H2A	0.988 (16)	C12—H12B	1.006 (17)
C3—C4	1.404 (2)	C12—H12C	0.998 (17)
C3—H1A	0.979 (14)	C13—H13A	0.984 (16)
C4—C5	1.3886 (18)	C13—H13B	0.967 (17)
C4—H4A	0.997 (15)	C13—H13C	1.009 (17)
C5—C6	1.3968 (18)	C14—H14A	1.000 (17)
C5—H5A	0.961 (15)	C14—H14B	1.001 (18)
C7—C12	1.5271 (19)	C14—H14C	1.010 (17)
C7—C8	1.5450 (18)		
			
C7—O1—H1O1	108.6 (12)	H8A—C8—H8B	106.5 (12)
C11—N1—C1	104.90 (11)	C13—C9—C14	109.35 (11)
C11—N2—C6	106.65 (10)	C13—C9—C10	109.98 (11)
C11—N2—C7	126.90 (10)	C14—C9—C10	109.00 (11)
C6—N2—C7	126.43 (10)	C13—C9—C8	113.17 (11)
N1—C1—C2	129.74 (12)	C14—C9—C8	108.46 (11)
N1—C1—C6	109.93 (11)	C10—C9—C8	106.78 (10)
C2—C1—C6	120.29 (12)	C11—C10—C9	110.96 (11)
C3—C2—C1	117.79 (12)	C11—C10—H10A	107.7 (8)
C3—C2—H2A	122.8 (9)	C9—C10—H10A	112.6 (8)
C1—C2—H2A	119.4 (9)	C11—C10—H10B	108.4 (9)
C2—C3—C4	121.33 (12)	C9—C10—H10B	109.2 (9)
C2—C3—H1A	119.5 (9)	H10A—C10—H10B	108.0 (12)
C4—C3—H1A	119.1 (9)	N1—C11—N2	113.35 (11)
C5—C4—C3	121.67 (13)	N1—C11—C10	125.66 (12)
C5—C4—H4A	118.4 (8)	N2—C11—C10	120.98 (11)
C3—C4—H4A	119.9 (8)	C7—C12—H12A	112.3 (10)
C4—C5—C6	116.79 (12)	C7—C12—H12B	106.4 (10)
C4—C5—H5A	119.5 (9)	H12A—C12—H12B	112.6 (14)
C6—C5—H5A	123.7 (9)	C7—C12—H12C	110.3 (10)
N2—C6—C5	132.67 (11)	H12A—C12—H12C	108.8 (14)
N2—C6—C1	105.13 (11)	H12B—C12—H12C	106.2 (14)
C5—C6—C1	122.06 (11)	C9—C13—H13A	112.9 (9)
O1—C7—N2	108.58 (10)	C9—C13—H13B	110.6 (9)
O1—C7—C12	106.80 (10)	H13A—C13—H13B	106.9 (13)
N2—C7—C12	109.84 (10)	C9—C13—H13C	111.5 (10)
O1—C7—C8	110.08 (10)	H13A—C13—H13C	107.1 (13)
N2—C7—C8	108.95 (10)	H13B—C13—H13C	107.4 (13)
C12—C7—C8	112.51 (11)	C9—C14—H14A	111.9 (9)
C9—C8—C7	118.09 (10)	C9—C14—H14B	109.3 (10)
C9—C8—H8A	108.9 (9)	H14A—C14—H14B	107.6 (13)
C7—C8—H8A	108.5 (9)	C9—C14—H14C	110.6 (10)
C9—C8—H8B	108.3 (9)	H14A—C14—H14C	105.5 (13)
C7—C8—H8B	106.0 (9)	H14B—C14—H14C	111.8 (14)
			
C11—N1—C1—C2	−177.80 (14)	C6—N2—C7—C12	−58.00 (16)
C11—N1—C1—C6	−0.14 (15)	C11—N2—C7—C8	0.12 (17)
N1—C1—C2—C3	176.20 (13)	C6—N2—C7—C8	178.34 (11)
C6—C1—C2—C3	−1.2 (2)	O1—C7—C8—C9	148.09 (11)
C1—C2—C3—C4	−1.3 (2)	N2—C7—C8—C9	29.14 (15)
C2—C3—C4—C5	2.2 (2)	C12—C7—C8—C9	−92.92 (14)
C3—C4—C5—C6	−0.5 (2)	C7—C8—C9—C13	64.20 (14)
C11—N2—C6—C5	173.80 (14)	C7—C8—C9—C14	−174.28 (11)
C7—N2—C6—C5	−4.7 (2)	C7—C8—C9—C10	−56.95 (15)
C11—N2—C6—C1	−1.75 (14)	C13—C9—C10—C11	−68.67 (15)
C7—N2—C6—C1	179.73 (11)	C14—C9—C10—C11	171.46 (11)
C4—C5—C6—N2	−176.96 (13)	C8—C9—C10—C11	54.49 (14)
C4—C5—C6—C1	−2.03 (19)	C1—N1—C11—N2	−1.04 (15)
N1—C1—C6—N2	1.20 (14)	C1—N1—C11—C10	177.57 (12)
C2—C1—C6—N2	179.11 (11)	C6—N2—C11—N1	1.82 (15)
N1—C1—C6—C5	−174.94 (11)	C7—N2—C11—N1	−179.67 (11)
C2—C1—C6—C5	3.0 (2)	C6—N2—C11—C10	−176.86 (12)
C11—N2—C7—O1	−119.77 (13)	C7—N2—C11—C10	1.64 (19)
C6—N2—C7—O1	58.45 (16)	C9—C10—C11—N1	150.69 (13)
C11—N2—C7—C12	123.78 (14)	C9—C10—C11—N2	−30.80 (17)

### Hydrogen-bond geometry (Å, °)

**Table:**

*D*—H···*A*	*D*—H	H···*A*	*D*···*A*	*D*—H···*A*
O1—H1O1···N1^i^	0.97 (2)	1.84 (2)	2.803 (2)	174 (2)
C5—H5A···O1^ii^	0.962 (15)	2.499 (15)	3.216 (2)	131.3 (11)

Symmetry codes: (i) −*x*+1, *y*+1/2, −*z*+1/2; (ii) −*x*+1, −*y*+1, −*z*.
